# Peritoneal Lymphomatosis: The Great Mimicker

**DOI:** 10.7759/cureus.14508

**Published:** 2021-04-15

**Authors:** Danial H Shaikh, Sudharsan Gongati, Syeda Hafsah Salman, Olga Alexandra Reyes, Sridhar Chilimuri

**Affiliations:** 1 Gastroenterology, BronxCare Health System, Bronx, USA; 2 Medicine/Internal Medicine, BronxCare Health System, Bronx, USA

**Keywords:** abdominal pain, ascites, peritoneum, omentum, lymphomatosis, carcinomatosis, lymphoma, chemotherapy, cancer

## Abstract

Peritoneal lymphomatosis (PL) is defined as intraperitoneal dissemination of lymphoma. Although rare, it is associated with high-grade lymphomas and can be easily mistaken for peritoneal carcinomatosis (PC) on imaging, which is a common condition associated with gastrointestinal and gynecological malignancies. Both PL and PC share similar radiographic features, however, they differ considerably in terms of prognosis and management. We present a case of a 58-year-old male with abdominal distention and ascites, initially reported as having PC on imaging. A subsequent peritoneal biopsy revealed PL secondary to a low-grade follicular lymphoma. Since PL responds well to chemotherapy, its prompt diagnosis and differentiation from PC helps avoid unnecessary surgery.

## Introduction

Neoplasms that involve the peritoneal cavity are secondary to the spread of primary malignancies from elsewhere. The peritoneum may become affected by three malignant cell lines: epithelial (carcinomatosis), mesenchymal (sarcomatosis), or lymphoid (lymphomatosis) [1].

Around 40% of lymphomas may have extra-nodal involvement (outside of the lymph nodes) and can involve almost any organ [2]. Peritoneal lymphomatosis (PL) is defined as the spread of lymphoma to the peritoneum, a rare occurrence, which is usually associated with high-grade lymphomas such as diffuse large B cell lymphoma (DLBCL) [3]. The peritoneal surface is composed of a fibro-fatty matrix devoid of any lymphoid tissue and as such the mechanism by which lymphomas spread to the peritoneum is unclear. One theory suggests the spread is via direct extension from the transverse mesocolon, the gastrocolic ligament, or the visceral peritoneal surface [4].

On imaging, PL can be easily mistaken for peritoneal carcinomatosis (PC), a common condition associated with disseminated malignancies, including gastrointestinal and gynecological malignancies. Peritoneal lymphomatosis responds well to chemotherapy and hence early diagnosis and differentiation from PC are imperative to avoid surgery. We present our experience of a patient who presented to our institution with abdominal pain and distention. Imaging revealed a pancreatic head mass with findings concerning for peritoneal carcinomatosis, however, a subsequent peritoneal biopsy revealed PL secondary to a low-grade follicular lymphoma.

## Case presentation

A 58-year-old male with no prior medical problems, who was leading an active lifestyle, presented to our emergency department with progressively worsening shortness of breath and abdominal distension for one month. His breathlessness had gradually worsened to the point where he felt winded even upon getting out of bed. He also reported intermittent abdominal discomfort associated with a decrease in appetite for a similar duration. Initially, the patient sought medical attention from his primary care physician and was started on treatment for community-acquired pneumonia without any significant improvement. He denied any fever, chills, night sweats, or weight loss. Review of systems was unremarkable including bowel habits, which were regular.

The patient had been a nonsmoker his entire life but had secondhand exposure to smoking from his wife. His alcohol consumption was limited only to social occasions and he denied any illicit drug use. He had no known allergies, nor did he take any daily medications. Family history was not significant for any malignancies. No recent travel was reported and by occupation, he was a tailor.

On examination, the patient was found lying comfortably in bed. Vital signs were significant for a pulse rate of 106 beats per minute, respiratory rate of 17 breaths per minute, and oxygen saturation of 98% on 2 liters of oxygen via nasal cannula. He was normotensive and afebrile. No cyanosis, clubbing, rash, or lymphadenopathy was noted. Respiratory examination revealed decreased air entry and dullness on percussion on the right side of the chest. His abdomen was distended, non-tender with a palpable liver and spleen, and he was noted to have shifting dullness. The rest of the examination was within normal limits. Laboratory data are summarized in Table 1.

**Table 1:** Laboratory Data

	Admission	Day 3	Day 8	Discharge
Hematology				
Hemoglobin (g/dL)	12.9	11.5	12	12.3
Mean corpuscular volume	85	82	84	85
White cell count (K/UdL)	3.6	4	4.3	3.5
Neutrophil%	73	72	66	68
Lymphocyte%	13.2	14.7	12.1	11.4
Platelets(k/ul)	250	208	210	215
Metabolic panel				
Sodium(mEq/L)	147	144	145	143
Potassium(mEq/L)	4.6	4.4	4.8	4.4
Bicarbonate(mEq/L)	28	27	25	30
chloride(mEq/L)	100	104	101	104
Blood Urea Nitrogen(mg/dL)	12	10	10	9
Creatinine(mg/dL)	1.1	1.2	1.3	1.1
Hepatic Panel				
Total protein(g/dL)	6.2	5.3	5.2	5.1
Albumin(g/dL)	4	3.3	3.1	3.2
Alanine aminotransferase(U/L)	11	11	10	7
Aspartate aminotransferase(U/L)	19	19	15	16
Alkaline phosphatase(U/L)	74	77	55	60
Total bilirubin/Direct bilirubin(mg/dL)	0.5/0.1	0.5/0.1	0.7/0.2	0.3/0.1
Prothrombin time (seconds)	13.3	12.3	12.3	13.1
Partial thromboplastin time (seconds)	29.9	30	32.6	41
Cancer antigen 19-9	0.7			
Human Immunodeficiency Virus	Negative			

**Figure 1:**
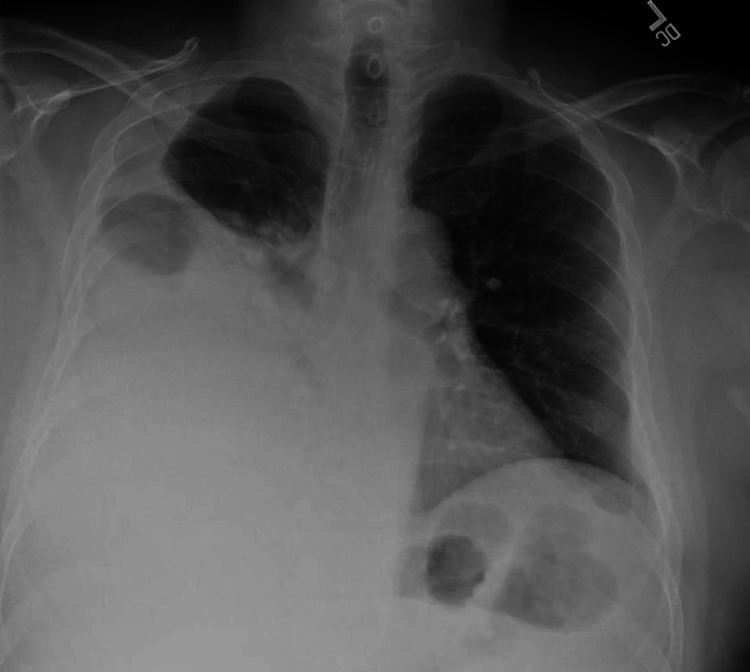
Chest x-ray demonstrating near complete opacification involving the mid to lower right lung along with moderate right side pleural effusion with minimal contralateral shift of the heart to the left side. The left lung is clear.

**Figure 2:**
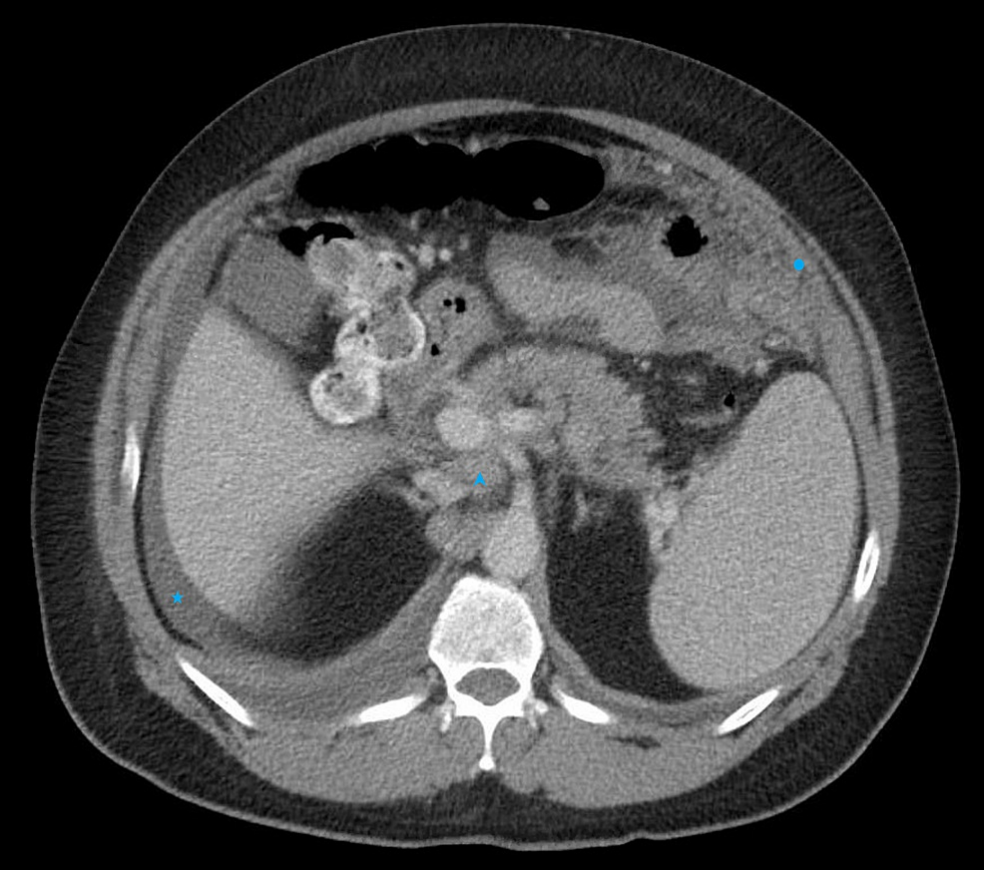
Contrast enhanced CT scan of the abdomen demonstrating bulky deposits (circle) in the left upper quadrant lateral to the transverse colon, diffuse retroperitoneal lymphadenopathy (arrowhead) seen posterior to the inferior vena cava, and mild perihepatic ascites (star).

**Figure 3:**
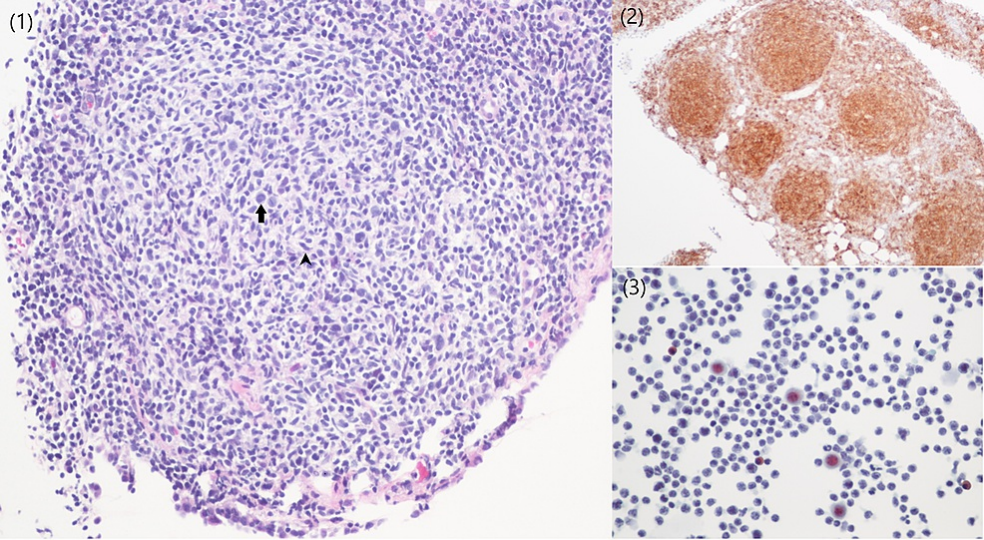
(1) Peritoneal biopsy showing a closely packed neoplastic follicle. The neoplastic follicle is composed of large centroblasts (arrow) surrounded by a variable number of centrocytes (arrowhead). (2) CD10 immunostaining is strongly immunoreactive in neoplastic follicles. (3) Pleural fluid with lymphomatous infiltrate.

Upon admission, a chest x-ray showed a near-complete opacity of the right hemithorax secondary to a large pleural effusion (Figure 1). A bedside thoracocentesis was performed and 600 milliliters of whitish milky pleural fluid was drained (Table 2). The pleural fluid analysis was consistent with chylothorax. A computed tomography (CT) scan of the abdomen and pelvis with contrast revealed a large soft tissue mass in the region of the pancreatic head with extensive diffuse lymphadenopathy and minimal perihepatic ascites (Figure 2). Further review of imaging revealed soft tissue deposits in the anterior abdomen and pelvis with peritoneal enhancement concerning for peritoneal carcinomatosis. The initial impression was pancreatic adenocarcinoma with peritoneal seeding. An oncology consultation was requested and a biopsy of the peritoneum was advised due to ease of access. An ultrasound-guided biopsy of the peritoneal lesion with analysis of the peritoneal fluid (Table 3) and a CT-guided placement of a right chest pleural drainage catheter was performed by interventional radiology. Analysis of the peritoneal pathology specimen revealed a mature B-cell neoplasm reported as a grade 2 follicular lymphoma (Figure 3), and immunophenotype stains revealed CD20+, CD79A+, CD10+, BCL-2+, BCL-6+, CD5-, CYCLIN D1-, CD3-, CD43-, AE1/AE3-. Cytology from the previously obtained pleural fluid also demonstrated lymphomatous infiltration. Due to persistent right pleural effusion, embolization of the thoracic duct was attempted without success. The patient was eventually transferred to a tertiary center under thoracic surgery service for further management of the high output chylothorax. He eventually was enrolled in the lymphoma program of the tertiary care center and started on chemotherapy.

**Table 2:** Pleural fluid analysis

White blood cell count (cells/mm^3^)	720
Segmental count	9%
Lymphocyte count	89%
Red blood cell count (cells/mm^3^)	2325
Albumin (g/dL)	3.2
Amylase (U/L)	50
Cholesterol (mg/dL)	115
Glucose (mg/dL)	106
Lactic Acid Dehydrogenase (U/L)	152
Total Protein (g/dL)	4.6
Triglyceride (mg/dL)	375

**Table 3:** Peritoneal fluid analysis

White blood cell count (cells/mm^3^)	389
Segmental count	5%
Lymphocyte count	93%
Red blood cell count (cells/mm^3^)	3875
Albumin (g/dL)	3.2
Amylase (U/L)	50
Cholesterol (mg/dL)	115
Glucose (mg/dL)	106
Lactic Acid Dehydrogenase (U/L)	152
Total Protein (g/dL)	4.6
Triglyceride (mg/dL)	13

## Discussion

Peritoneal lymphomatosis and carcinomatosis are among a host of pathologies that may involve the peritoneum. These include peritoneal sarcomatosis (from gastrointestinal stromal tumors, leiomyosarcomas, or liposarcomas), tuberculosis, and pseudomyxoma peritonei [5].

Peritoneal involvement from the secondary spread of tumors is much more common than a primary peritoneal tumor. Primary tumors such as peritoneal mesothelioma and mesenteric sarcomas are rare, whereas diffuse peritoneal and omental seeding due to malignant diseases of the ovaries, colon, and stomach is a very common phenomenon [6]. PC has been identified in almost 15% of patients with colorectal cancer and almost 40% of patients with stage 2 or 3 gastric cancer on abdominal exploration [7].

Owing to its rare nature, PL receives significantly less attention than PC, and only a handful of reports are found in the literature [3,8]. In a case series of 101 patients with malignancy-associated ascites, only 8% of cases of lymphoma were reported [8]. An autopsy series of 322 patients with non-Hodgkin’s lymphoma (NHL) reported omental involvement by lymphoma in only 64 patients (20%) [6].

Clinical manifestations of PL include abdominal distension, abdominal pain, fever, emaciation, or malaise. Smooth thickening of the peritoneum and a diffuse mass involving the peritoneal layer of the bowel due to lymphoma is reported. Peritoneal lymphomatosis can manifest as discrete nodules involving the peritoneum and as exudative ascites that is high in protein [2,9,10]. The ascites, however, is mild to moderate and less than that associated with PC [6].

PL has common imaging (CT or MRI) features with PC including pleural effusion, ascites, anasarca, omental caking, peritoneal thickening, and serosal disease. Omental caking is the appearance of fine nodular or large bulky soft tissue deposits involving the peritoneum secondary to malignancy. The large subtype is associated with lymphomatous spread [1].

Findings distinctive to PL include a greater burden of lymphadenopathy with a higher number of involved nodes, larger associated nodules, and more frequent hepatosplenomegaly [11]. Another CT imaging finding reported in patients with mesenteric lymphoma is the ‘sandwich sign’, formed by mesenteric soft tissue masses that embed and encircle the mesenteric vessels and perivascular fat [12]. Ultrasound imaging may also aid in the diagnosis of peritoneal lymphoma. The ‘fish scale sign’ seen on ultrasound, is the thickened omentum due to lymphomatous infiltration that results in hyperechoic areas superimposed on a hypoechoic background [13].

Fluorodeoxyglucose-positron emission tomography (FDG-PET)/CT helps in better staging and monitoring the treatment response and aids in the early detection of disease recurrence. In subtle cases, the sensitivity of detection of peritoneal involvement and extranodal disease is better than that of CT. This imaging also helps in choosing an appropriate biopsy site and increases the chance of detection of early disease. Its sensitivity and specificity for detecting peritoneal seeding reach as high as 100% and 97%, respectively [11]. 

Diagnosing lymphomas in effusions and/or ascites can be challenging, especially if the lymph node architecture is not available for analyses. Studies like flow cytometry and immunohistochemical staining aid in making an accurate diagnosis and help in identifying the molecular characteristics of the lymphoma and its immunophenotype, which dictate treatment decisions [14]. Nevertheless, histology remains the gold standard for diagnosis [15].

The patient described in the case initially presented with shortness of breath. Chest imaging revealed bilateral pleural effusions. The pleural fluid analysis showed elevated cholesterol and triglyceride levels, suggestive of chylothorax. The major cause of non-traumatic chylothorax is malignancy. Most cases of malignancy-associated chylothorax occur in patients with advanced disease [16,17]. CT scan of the abdomen and pelvis revealed a soft tissue density in the pancreatic head with extensive para-aortic, epi-phrenic, and peri-celiac lymphadenopathy and splenomegaly. Primary pancreatic lymphoma consists of less than 0.5% of pancreatic tumors. Secondary involvement of the pancreas from adjacent organs or peri-pancreatic lymph nodes is more common [18,19].

**Figure 4:**
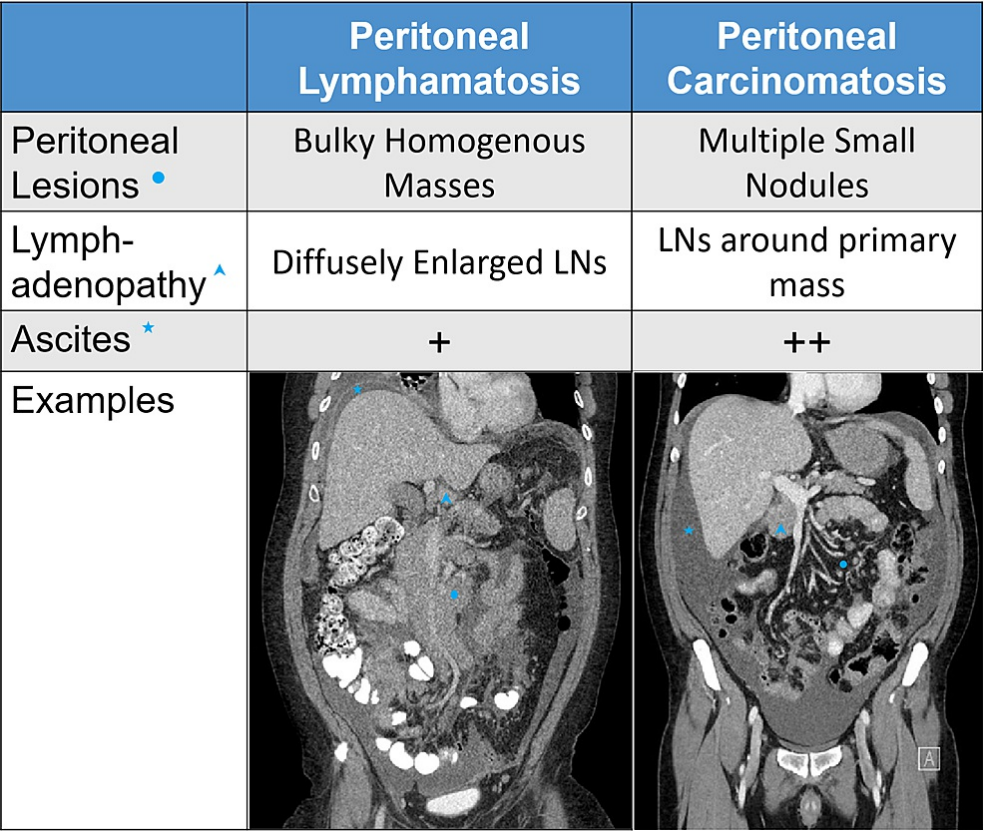
Comparison of the imaging characteristics between peritoneal lymphomatosis and peritoneal carcinomatosis Adapted from Cabral et al. [1] LN: lymph node

Upon further review of this patient’s CT imaging, soft tissue deposits in the anterior pelvis with peritoneal enhancement concerning for PC were present. A diagnosis of PL based on imaging can be difficult to make considering its rarity and its similarity to peritoneal carcinomatosis on imaging (Figure 4), hence a presumptive diagnosis should be confirmed with a biopsy. Nevertheless, CT remains the first choice of imaging for the workup of suspected peritoneal lymphomatosis [20].

Patients with a pancreatic mass and peritoneal metastasis are often ceded to palliative care, however, a diagnosis of lymphoma will change the management. Treatment of PL is non-surgical as dramatic improvement can be seen with chemotherapy. Upon suspicion, early communication with a specialist regarding the possibility of lymphoma may facilitate earlier diagnosis and treatment, thereby preventing unnecessary surgery.

## Conclusions

Our patient's imaging findings had a presumptive diagnosis of peritoneal carcinomatosis; however, biopsy results were consistent with a mature B cell neoplasm characterized as a grade 2 follicular lymphoma. The patient was transferred to a specialized lymphoma center for further evaluation and treatment. As peritoneal lymphomatosis responds well to chemotherapy, we advocate for earlier detection based on subtle yet distinct imaging findings, allowing for its evaluation and treatment in a timely manner, while avoiding any unnecessary abdominal surgery.

## References

[1] Cabral FC, Krajewski KM, Kim KW, Ramaiya NH, Jagannathan JP (2013). Peritoneal lymphomatosis: CT and PET/CT findings and how to differentiate between carcinomatosis and sarcomatosis. Cancer Imaging.

[2] Lee WK, Lau EW, Duddalwar VA, Stanley AJ, Ho YY (2008). Abdominal manifestations of extranodal lymphoma: spectrum of imaging findings. AJR Am J Roentgenol.

[3] Weng SC, Wu CY (2008). Lymphoma presenting as peritoneal lymphomatosis with ascites. J Chin Med Assoc.

[4] Sharifah MI, Zamzami NA, Rafeah TN (2011). Diffuse peritoneal lymphomatosis simulating peritoneal carcinomatosis. Med J Malaysia.

[5] O'Neill AC, Shinagare AB, Rosenthal MH, Tirumani SH, Jagannathan JP, Ramaiya NH (2014). Differences in CT features of peritoneal carcinomatosis, sarcomatosis, and lymphomatosis: retrospective analysis of 122 cases at a tertiary cancer institution. Clin Radiol.

[6] Karaosmanoglu D, Karcaaltincaba M, Oguz B, Akata D, Ozmen M, Akhan O (2009). CT findings of lymphoma with peritoneal, omental and mesenteric involvement: peritoneal lymphomatosis. Eur J Radiol.

[7] Coccolini F, Gheza F, Lotti M (2013). Peritoneal carcinomatosis. World J Gastroenterol.

[8] Runyon BA, Hoefs JC (1986). Peritoneal lymphomatosis with ascites. A characterization. Arch Intern Med.

[9] Levy AD, Shaw JC, Sobin LH (2009). Secondary tumors and tumorlike lesions of the peritoneal cavity: imaging features with pathologic correlation. Radiographics.

[10] Lynch MA, Cho KC, Jeffrey RB Jr, Alterman DD, Federle MP (1988). CT of peritoneal lymphomatosis. AJR Am J Roentgenol.

[11] Dirisamer A, Schima W, Heinisch M, Weber M, Lehner HP, Haller J, Langsteger W (2009). Detection of histologically proven peritoneal carcinomatosis with fused 18F-FDG-PET/MDCT. Eur J Radiol.

[12] Hardy SM (2003). The sandwich sign. Radiology.

[13] Que Y, Wang X (2015). Sonography of peritoneal lymphomatosis: some new and different findings. Ultrasound Q.

[14] Solomon JP, Arcila ME (2020). Molecular diagnostics of non-Hodgkin lymphoma. Cancer J.

[15] Perry AM, Mitrovic Z, Chan WC (2012). Biological prognostic markers in diffuse large B-cell lymphoma. Cancer Control.

[16] Doerr CH, Allen MS, Nichols FC 3rd, Ryu JH (2005). Etiology of chylothorax in 203 patients. Mayo Clin Proc.

[17] Gomes AO, Ribeiro S, Neves J, Mendonça T (2015). Uncommon aetiologies of chylothorax: superior vena cava syndrome and thoracic aortic aneurysm. Clin Respir J.

[18] Malin ES, Toomey CE, Ono J (2010). Primary pancreatic lymphoma. Blood.

[19] Saif MW (2006). Primary pancreatic lymphomas. JOP.

[20] Zelenetz AD, Gordon LI, Abramson JS (2019). NCCN guidelines insights: B-cell lymphomas, version 3.2019. J Natl Compr Canc Netw.

